# A meta-analysis of acupuncture at the sphenopalatine ganglion for the treatment of allergic rhinitis

**DOI:** 10.3389/falgy.2026.1780330

**Published:** 2026-05-13

**Authors:** Qiang Liu, Weiying Zhu, YongDong Sun, Long Chen

**Affiliations:** Department of Otolaryngology Head and Neck Surgery, The Affiliated Traditional Chinese Medicine Hospital of Southwest Medical University, Luzhou, Sichuan, China

**Keywords:** allergic rhinitis, overall response rate, RQLQ, sphenopalatine ganglion, systematic review and meta-analysis, TNSS

## Abstract

**Background:**

Allergic rhinitis (AR) imposes a significant global health burden. While conventional treatments include medications and immunotherapy, acupuncture at the sphenopalatine ganglion (SPG) has emerged as a promising alternative. This meta-analysis evaluates the efficacy of SPG acupuncture for AR.

**Methods:**

We searched CNKI, PubMed, Web of Science, SinMed, and Cochrane Library (inception to May 2024) for RCTs comparing SPG acupuncture against controls (conventional acupuncture/sham/medications). Primary outcomes were Rhinoconjunctivitis Quality of Life Questionnaire (RQLQ) change, Total Nasal Symptom Score (TNSS) change, and total effective rate. Risk of bias was assessed using Cochrane criteria. Meta-analyses used random/fixed models in RevMan 5.3; evidence quality was graded via GRADE.

**Results:**

Eleven RCTs (= 732) were analyzed. SPG acupuncture significantly: Improved RQLQ (MD = 7.63, 95% CI: 2.81 to 12.45, *P* = 0.002; low-quality evidence).Reduced TNSS (MD = 2.23, 95% CI: 0.92 to 3.55, *P* = 0.0009; low-quality evidence).Increased total effective rate ((RR = 1.17, 95% CI: 1.06 to 1.29, *P* = 0.002; moderate-quality evidence).No IgE improvement was observed (*P* = 0.23). Heterogeneity was high for RQLQ (I^2^ = 89%) and TNSS (I^2^ = 83%).

**Conclusion:**

Eleven RCTs with a total of 732 participants were analyzed. SPG acupuncture improves quality of life, nasal symptoms, and clinical efficacy in AR patients vs. conventional treatments, though evidence quality remains low to moderate. It represents a viable adjunct therapy, warranting further high-quality trials.

**SYSTEMATIC REVIEW REGISTRATION:**

https://www.crd.york.ac.uk/prospero/

## 1 Introduction

Allergic rhinitis (AR) is a type I allergic disease caused by exposure of atopic individuals to specific allergens (1, 2)。At present, the main treatment options are drug therapy and immunotherapy, such as nasal glucocorticoids, oral antihistamines, anti-leukotrienes, allergen-specific immunotherapy, etc (3, 4)。A large number of studies have shown that acupuncture treatment has the advantages of relieving symptoms quickly, shortening the duration of attacks and improving the quality of life (5, 6).

Acupuncture of sphenopalatine ganglion is a special acupuncture method created by Professor Xinwu Li in the 1960s (7), which is a combination of traditional acupuncture and anatomy. The Sphenopalatine ganglion, the largest parasympathetic ganglion in the head, plays a pivotal role in regulating the secretory activity and sensory function of the lacrimal gland, nasopharynx, and nasal mucosa. It is widely recognized to be closely associated with the neuro-related pathophysiological mechanisms underlying allergic rhinitis (8). As a targeted intervention, PPG acupuncture is clinically employed for the management of allergic rhinitis. To optimize therapeutic outcomes, PPG acupuncture is frequently combined with other therapeutic modalities in clinical practice. The combination therapy of PPG acupuncture and traditional acupuncture has demonstrated more rapid, potent, and sustained efficacy in symptom alleviation, with a significantly higher response rate at week 8 compared to monotherapy with medications or traditional acupuncture alone (9). Current clinical evidence supports PPG acupuncture as an effective therapeutic approach for allergic rhinitis. Whether administered as a standalone treatment, in combination with traditional acupuncture, or in the form of ultrasound-guided block, this therapy can significantly relieve nasal symptoms and enhance patients’ quality of life, thereby providing a critical non-pharmacological alternative for patients with refractory allergic rhinitis or those intolerant to conventional pharmacotherapy.

In recent years, the literature on its clinical application has gradually increased, but the research quality gap is large and systematic summary is lacking. In this paper, relevant literatures in major mainstream databases were retrieved, original research data were included through screening and quality control, and secondary comprehensive analysis and evaluation were conducted. The efficacy was evaluated from a more objective and standard perspective with the help of meta-analysis in evidence-based medicine, providing reliable evidence-based basis for clinical practice.

## 2 Materials and methods

### 2.1 General information

This review adheres strictly to the criteria stipulated in the Preferred Reporting Items for Systematic Reviews and Meta-Analyses (PRISMA) 2020 statement. The protocol has been duly registered with PROSPERO, bearing the registration number CRD42024546310.

### 2.2 Search terms

The subjects (allergic rhinitis) and intervention methods (acupuncture at sphenopalatine ganglion) were used as the search terms, and the search terms were found from the literature and diagnosis and treatment guidelines by the method of subject words + free words. The Chinese search terms are:变应性鼻炎、过敏性鼻炎、鼻鼽、针刺, 针灸、蝶腭神经节、翼腭神经节、新吾穴、蝶腭穴。English search terms are:allergicrhinitis、AllergicRhinitides、anaphylacticrhinitis、rhinallergosis、anaphylacticrhinitis、hypersensitiverhinitis、allergiccoryza、allergic-rhintis; acupuncture、Pharmacopuncture、PharmacoacupunctureTreatment、PharmacoacupunctureTherapy、Acupotomy、Acupotomies、acumox; SphenopalatineGanglion、PterygopalatineGanglion、sphe-nomaxillaryganglion、nasalganglion、Meckelganglion、sphenoma-xillaryganglion、ganglionsphenopalatinum。

### 2.3 Search strategy

The Chinese databases used included SinMed and CNKI. The English databases used included: PubMed, WebofScience, Cochranelibrary. The time was selected from the establishment of the database to May 31, 2024, and the language of the literature was limited to Chinese or English. EndnoteX9 software was used to save all the detected literature and record the search strategy used.

### 2.4 Inclusion criteria and exclusion criteria

#### 2.4.1 Inclusion criteria

**Figure 1:**
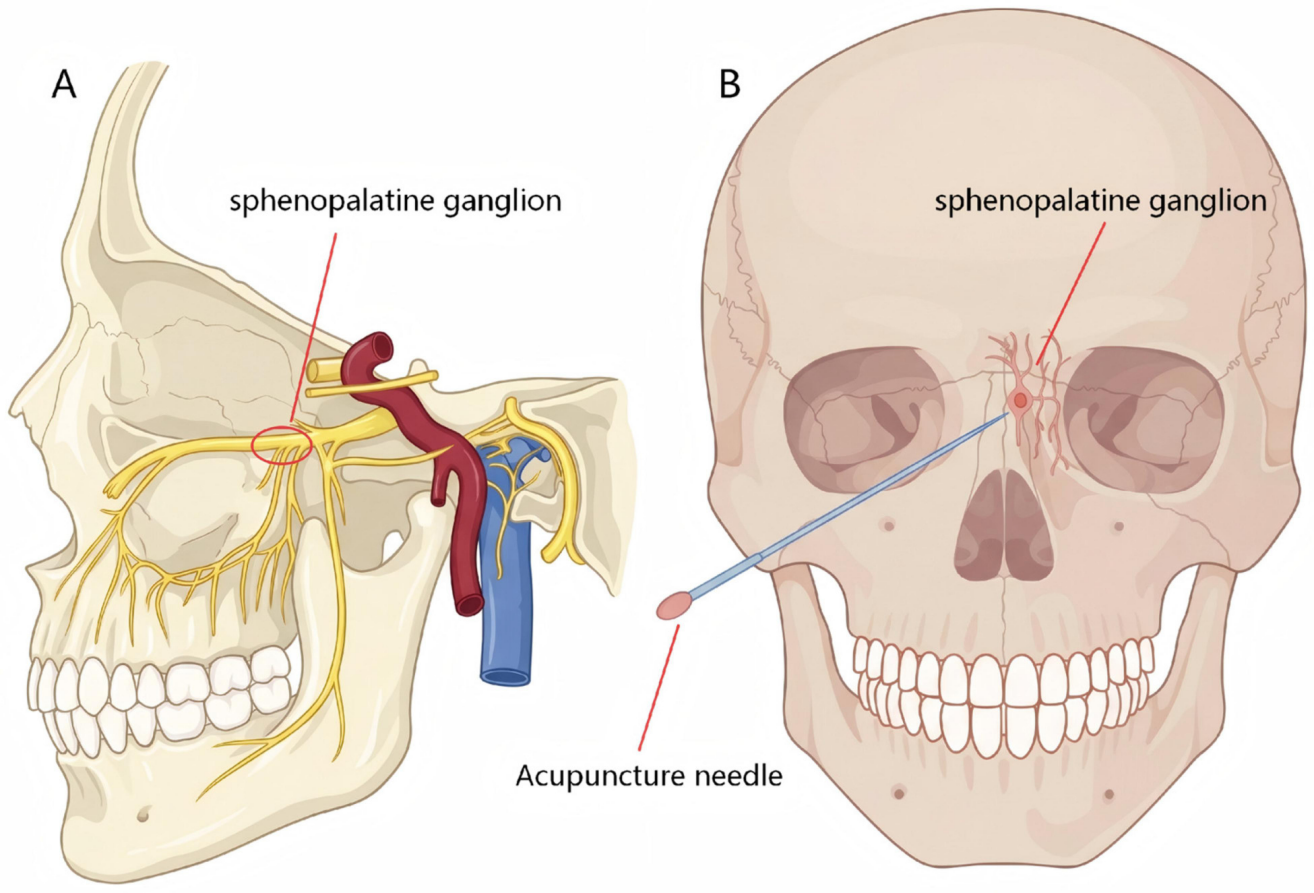
**(A)** Sagittal simplified diagram of the pterygopalatine ganglion. **(B)** Simplified coronal view of acupuncture needle insertion into the sphenopalatine ganglion.

1. Subjects: The clinical diagnosis of allergic rhinitis should meet the diagnostic criteria of allergic rhinitis in the “Principles and Recommendations for the diagnosis and Treatment of Allergic Rhinitis” (2004, Lanzhou) (10).
2. Intervention: The intervention group was treated with acupuncture at sphenopalatine ganglion (SPG group), as shown in the Figure 1. The specific method was as follows: the patient was in a sitting position, the head was tilted back later, and the body surface projection was equivalent to the intersection of the line from the lateral canthus to the mandibular Angle and the outer and lower edge of the zygomatic arch, as the needle entry point. The 0.35mm × 60 mm filiform needle was inserted slowly with the needle tip toward the contralateral temporal region, and the depth of the needle was 55 mm. The patient could feel the sensation of electric shock or numbness, which was transmitted to the nose or lip, and there was a water-spraying sensation in the nose. The needle can be inserted at the same time on both sides, or alternatively on the left and right sides. There was no special limitation on the frequency of acupuncture.
3. Control measures: the control group was treated with conventional acupuncture, sham acupuncture or medication recommended by clinical guidelines. Conventional acupuncture refers to conventional filiform needling or lifting of the needle. The main acupoints are Yingxiang (GV 14), Yintang (GV 29), Hegu (LI 4) and Fengchi (GB 20). Sham acupuncture was the same as the intervention group except that the depth was less than 20 mm. Medications included nasal corticosteroids, oral antihistamines, nasal antihistamines, and oral antileukotrienes. The course of treatment was more than 2 weeks.
4. Outcome indicators included one of ①, ②, and ③. ① Rhinoconjunctivitis quality of life questionnaire (11) (RQLQ): the RQLQ score before treatment -RQLQ score after treatment。② Difference in total nasal symptom score（TNSS）(10) before and after treatment。The symptom grading scoring standard included nasal itching, sneezing, nasal obstruction and runny nose, and was scored according to the severity of the attack. ③ Total effective rate (12): According to the rhinitis symptoms and signs score, the patients were divided into markedly effective, effective and ineffective. The calculation method is as in equation:
  $$\begin{array}{rl} & \mathrm{Total}\;\mathrm{effective}\;\mathrm{rate}=(\mathrm{significant}\;\mathrm{effective}\;\mathrm{cases}\;+\\ & \;\mathrm{number}\;\mathrm{of}\;\mathrm{effective}\;\mathrm{cases})/\mathrm{total}\;\mathrm{number}\;\mathrm{of}\;\mathrm{cases}\times 100\text{\%}. \end{array}$$
5. Type of study: Randomized controlled trial.

#### 2.4.2 Exclusion criteria

① The articles with lower quality completeness were excluded if they were published repeatedly or with the same data.② Intervention combination with traditional Chinese medicine, western medicine or other surgical treatment methods.③ Studies of disease group/non-disease group RCT, can not obtain full text literature.④ Low quality literature with high risk of bias.

### 2.5 Data extraction and quality assessment

All tests were performed independently by two investigators and cross-checked. EndnoteX9 software was used to remove duplicate literature. The first screening: according to the inclusion and exclusion criteria, the title and abstract were screened, and the reasons for exclusion were recorded. The second screening: the full text was read in detail, the articles that did not meet the standards of outcome indicators were excluded and the reasons were recorded. Third screening: low-quality literature was excluded.

The Cochrane (13) risk bias assessment tool was used to evaluate the risk of bias of the included literature that met the inclusion criteria. Low risk, unclear, and high risk were evaluated according to the description of the research methods in the included literature. If all six aspects of the risk of bias are assessed as low risk, the research evidence is considered to have a low risk grade; If the risk of bias in any one aspect is assessed as high risk, the research evidence is considered to have a high risk level. If the risk of bias of any of the above six aspects was not assessed as high, but the risk of bias of some aspects was assessed as uncertain or there was a lack of relevant information, the research evidence was considered to be at the risk of uncertainty. Disagreements were resolved by discussion or by requesting a third researcher to arbitrate the decision.

### 2.6 Data extraction and processing

Data were extracted from the included literatures, and a feature table was made, including title, author, publication time, literature source, paper type, age, gender, intervention measures, sample size, treatment course, and outcome indicators. Data were extracted from the included literatures, and a feature table was made, including title, author, publication time, literature source, paper type, age, gender, intervention measures, sample size, treatment course, and outcome indicators. For articles that only present the mean and standard deviation of RQLQ or TNSS before and after treatment, the change in standard deviation is calculated using the following equation:

$$\begin{array}{rl} SD_{\mathrm{change}} & =\mathrm{square}\;\mathrm{root}\;[{\left(SD_{\mathrm{baseline}}\right)}^{2}+{\left(SD_{\mathrm{postintervention}}\right)}^{2}\\ & \;-(2R\times SD_{\mathrm{baseline}}\times SD_{\mathrm{postintervention}})] \end{array}$$

Where R is 0.5. Authors were contacted in cases of missing data to obtain complete data if possible.

### 2.7 Statistical analysis

RevMan5.3 software was used to analyze the included data. Mean difference (MD) and odds ratio (OR) with 95% confidence interval (CI) were used for statistical analysis. If I^2^ ≥ 50%, *P* ≤ 0.1, indicating large heterogeneity, using the random effects model; otherwise, indicating small heterogeneity, using the fixed effects model. *P* < 0.05 was considered statistically significant.

If more than 10 studies were included, funnel plot was used to observe publication bias. RevMan5.3 software was used to make a funnel plot with RQLQ score as the index to observe whether there was publication bias. If the funnel plot was symmetrical, there was no publication bias. If the figures are asymmetric, publication bias exists. The Egger^,^s and Begg's tests were used to test for publication bias when the figures could not be judged.

## 3 Results

### 3.1 Literature search and screening results

**Figure 2:**
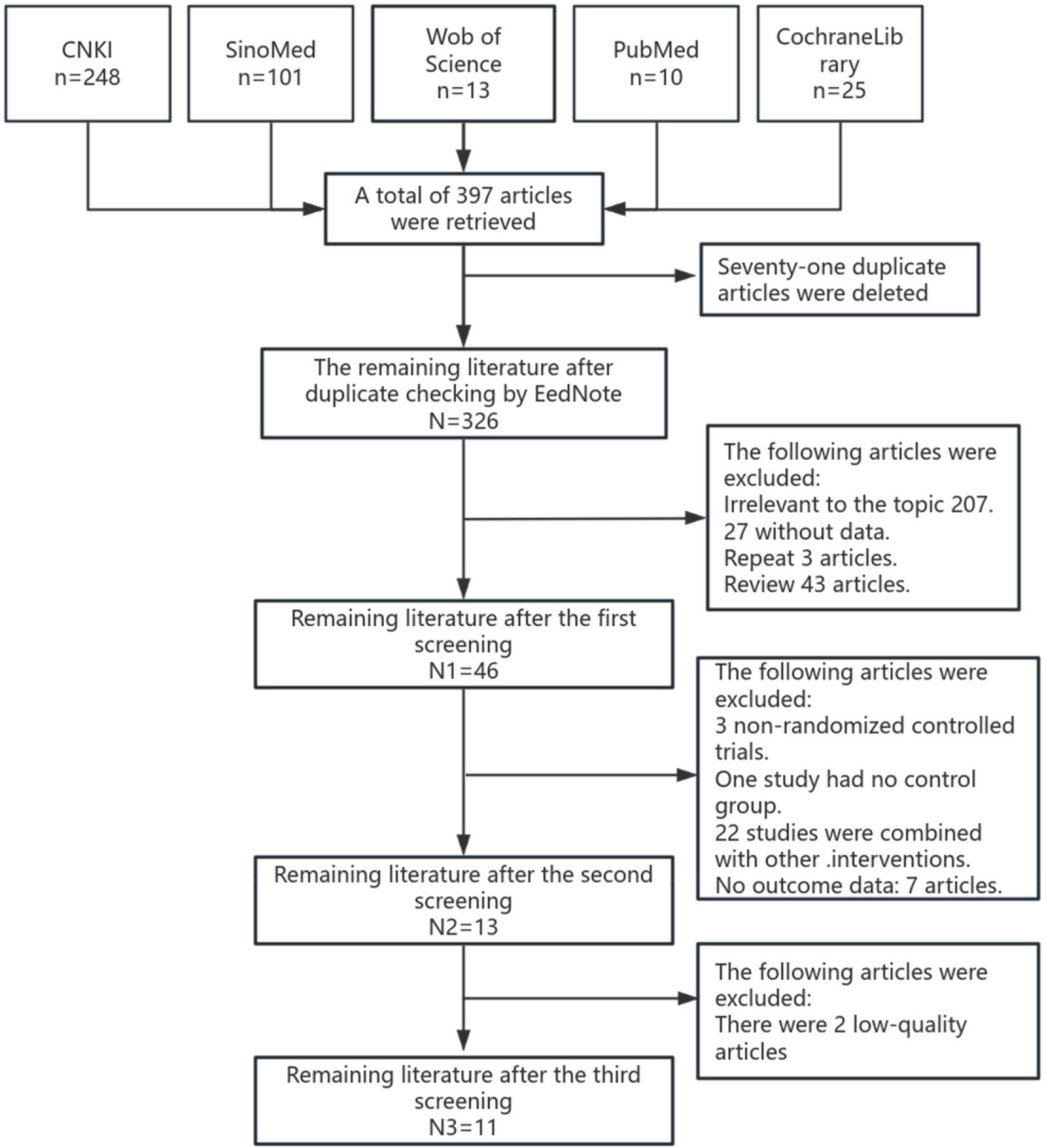
Flow chart of literature screening.

397 kinds of literature were retrieved, and 326 remained after EndNoteX9 software eliminated duplicate literature. After the initial screening, 46 literatures, including 39 Chinese and 7 English, were included in the second screening. After the second screening, 13 articles were obtained, and 11 were finally included. The screening procedure is shown in Figure 2.

### 3.2 Results of literature bias risk assessment

**Figure 3:**
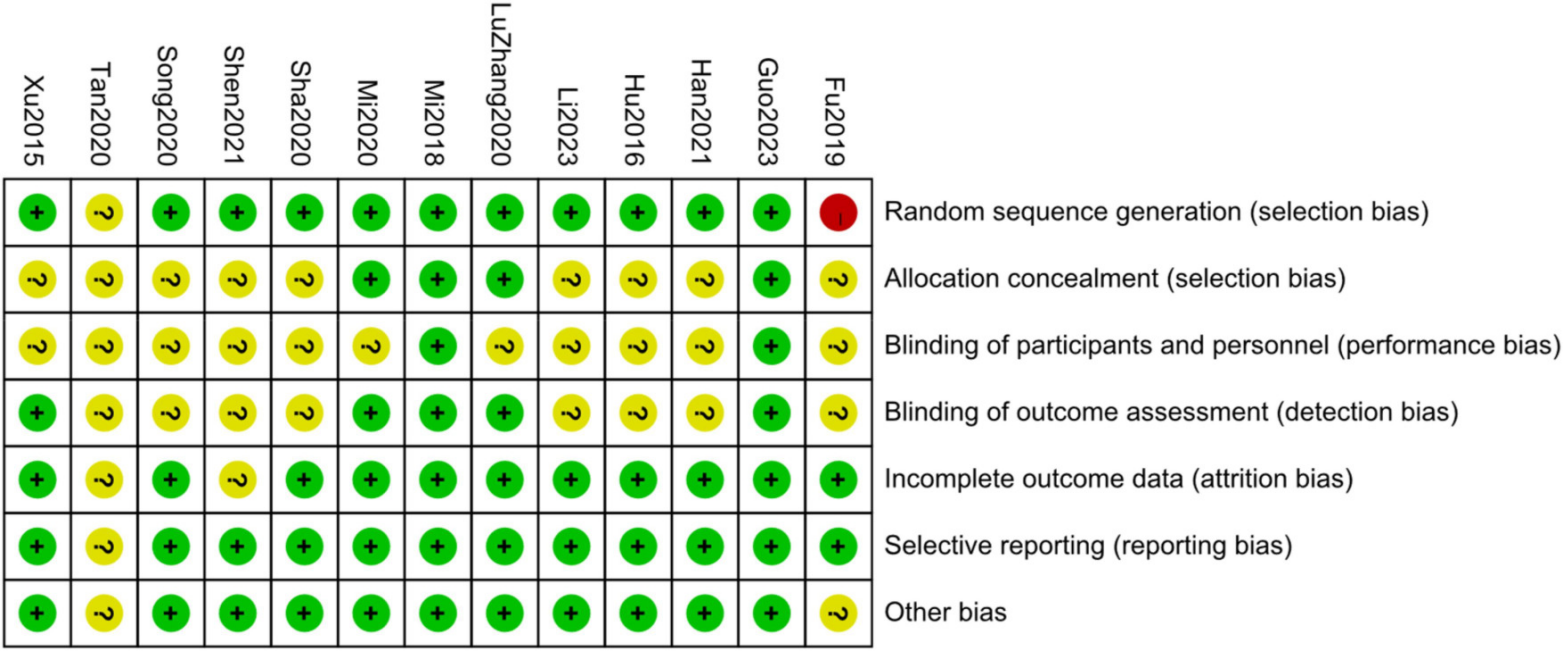
Summary chart of risk assessment of 13 articles.

**Figure 4:**
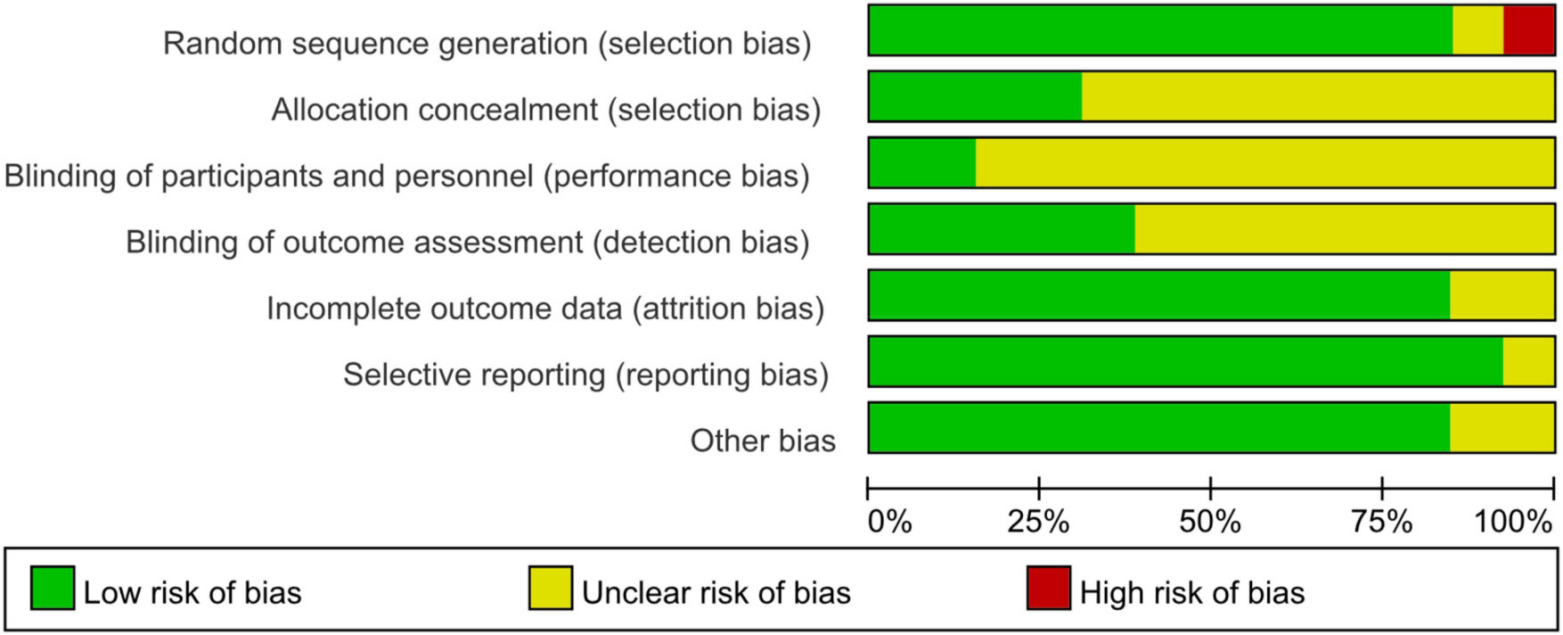
Risk assessment charts of 7 items in 13 literatures.

According to the evaluation method provided by the Cochrane handbook, Guo2023 (14)、Mi2018 (15) were low-risk literatures. Hu2017 (33)、Sha2020 (16)、Xu2015 (17)、Mi2020 (18)、Han2021 (19)、ZhangL2020 (20)、Shen2021 (21)、Li2023 (22)、Song2020 (23) belong to missing part of the information but no high wind Risks covered. Tan2020 (24) indicates that the risk level cannot be determined. Fu2019 (25) is a high-risk literature. According to the evaluation results, Fu2019 and Tan2020 were excluded because of their high risk of bias. RevMan5.3 software was used to draw the risk of bias assessment chart, and the assessment results are shown in Figures 3, 4. After excluding high-risk literatures, 11 literatures were finally included.

### 3.3 Description of the basic characteristics of the included literatures

A total of 11 studies with 732 samples were included. Among them, 7 were controlled by conventional acupuncture and sham acupuncture, 2 were controlled by conventional western medication, and 1 was controlled by both conventional acupuncture and conventional western medication. RQLQ score was used as the outcome index in 10 studies, TNSS was used as the outcome index in 7 studies, total effective rate was used as the outcome index in 6 studies, and IgE was used as the outcome index in 3 studies (Table 1).

**Table 1:** Basic characteristics of the included literature.

AUTHOR AND YEAR	LITERATURE SOURCES	STUDY DESIGN	PATIENTS (N) SPG/NON-SPG	CONTROL GROUP INTERVENTIONS	OUTCOME MEASURES
1. GUO 2023 (14)	Journal of Traditional Chinese Medicine	RCT	19/18	Traditional acupuncture	RQLQ、TNSS
2. HU2017 (33)	Chinese Journal of InformationonTraditional Chinese Medicine	RCT	32/33	Traditional acupuncture	RQLQ、TNSS、Total effective rate
3. SHA2020 (16)	Nanjing University Of Chinese Medicine	RCT	34/34	Loratadine	RQLQ、TNSS、Total effective rate
4. XU2015 (17)	Beijing University Of Chinese Medicine	RCT	35/34	Traditional acupuncture	RQLQ、TNSS
5. MI 2020 (18)	MedAcupunct	RCT	33/36/35	1. Traditional acupuncture"	
2. Budesonide nasal spray	RQLQ				
6. MI 2018 (15)	Trials	RCT	30/31	Sham acupuncture	RQLQ
7. HAN2021 (19)	Journal of Clinical Acupuncture and Moxibustion	RCT	36/36	Traditional acupuncture	RQLQ、TNSS、IgE、Total effective rate
8. ZHANG 2020 (20)	EvidBased Complement AlternatMed	RCT	48/48	Traditional acupuncture	RQLQ、TNSS
9. SHEN 2021 (21)	Chinese Journal of OtorhinolaryngologyinIntegrative Medicine	RCT	30/30	Azelastine hydrochloride nasal spray	RQLQ、TNSS、Total effective rate
10. LI2023 (22)	Guang xi Medical Journal	RCT	50/50	Traditional acupuncture	RQLQ、IgE、Total effective rate
11. SONG2020 (23)	China Medicine and Pharmacy	RCT	32/32	Loratadine、Budesonide nasal spray	IgE、Total effective rate

### 3.4 Results of meta-analysis

#### 3.4.1 RQLQ score change value analysis

**Figure 5:**
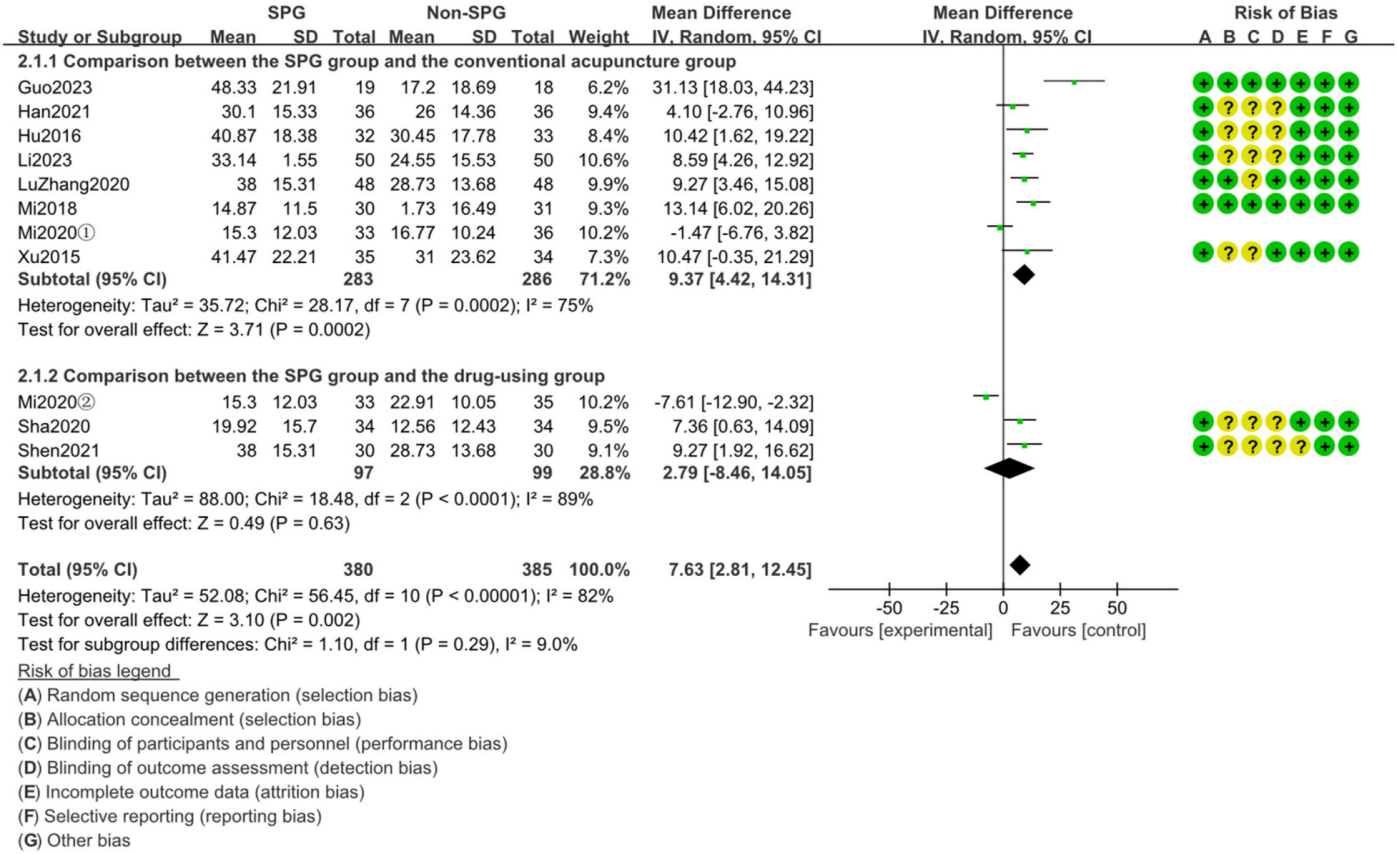
The analysis results of the difference of RQLQ (rhinoconjunctivitis quality of life questionnaire) scores before and after treatment between the SPG group and the control group.

A total of 10 studies were included for the analysis of RQLQ score change, where RQLQ score is the difference of the patient's score before treatment minus the score after treatment. Mi2020 included multiple control groups, so there were 11 groups of data in the analysis results, with large heterogeneity (I^2^ = 88.9%, *P* < 0.00001) and poor homogeneity. Using the random effect size model, the pooled effect size MD = 7.63,95%CI[2.81,12.45],Z = 3.05(*p* = 0.002), and the 95%CI did not cross the null line, suggesting that the statistical results were different. In the treatment of allergic rhinitis, acupuncture of sphenopalatine ganglion was superior to the intervention measures of the control group in improving the RQLQ score (*p* = 0.002). In the subgroup analysis of different intervention measures of the control group, the statistical difference was more obvious when acupuncture of sphenopalatine ganglion was compared with ordinary acupuncture (*p* = 0.0003), as shown in Figure 5.

#### 3.4.2 Analysis of TNSS score changes

**Figure 6:**
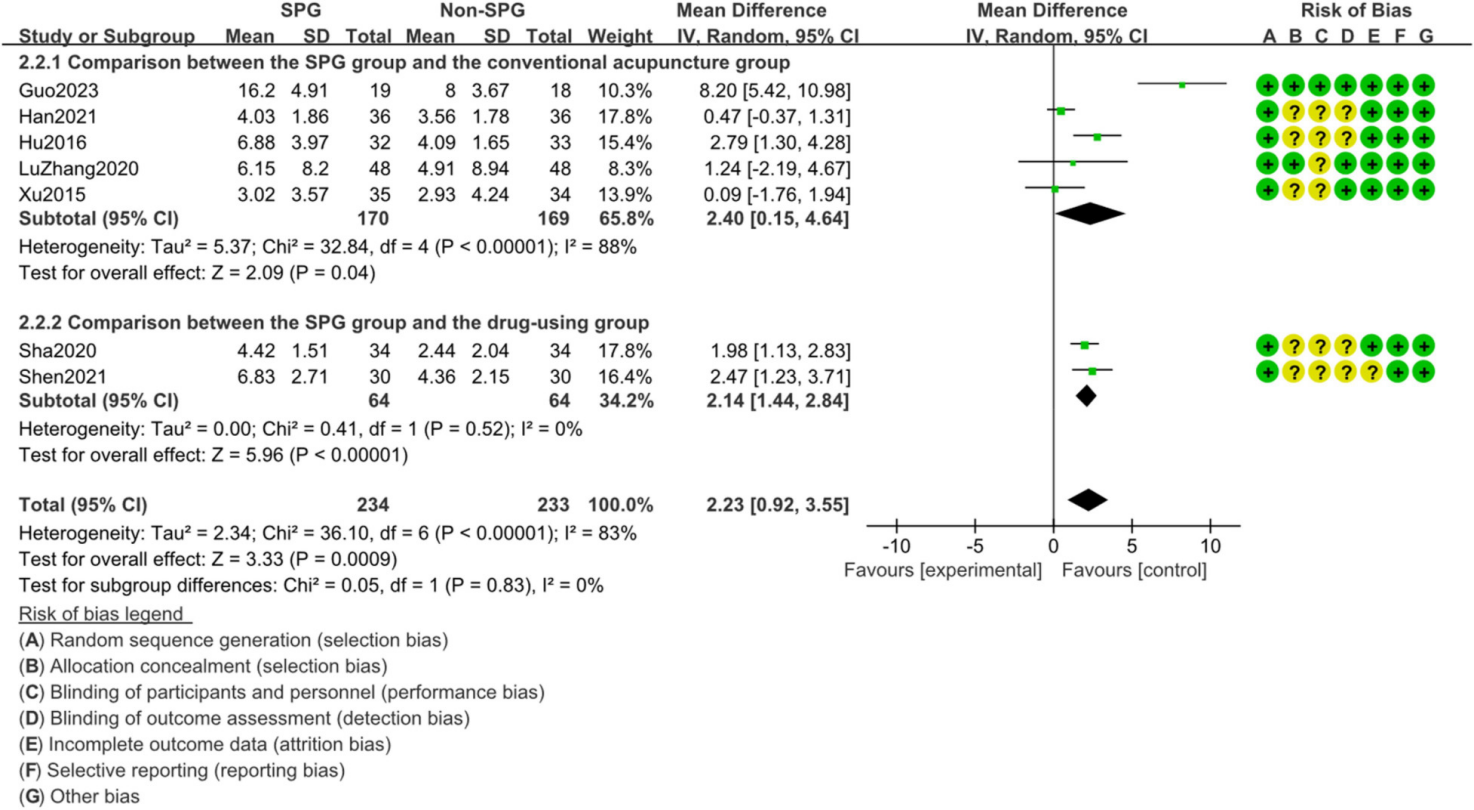
Results of the analysis of the difference in TNSS (total nasal symptom score) before and after treatment. (SPG: acupuncture of the pterygopalatine ganglion group).

A total of 7 studies were included for the analysis of changes in rhinitis symptom scores. The heterogeneity test (I^2^ = 83%, *P* < 0.00001) showed heterogeneity, so a random effect model was used. The combined effect size was MD = 2.23,95%CI[0.92,3.55],Z = 3.33(*p* = 0.0009),95%CI did not cross the null line. This suggests a difference in the statistical results. Acupuncture of sphenopalatine ganglion significantly improved the symptom scores of rhinitis compared with the control group (*p* < 0.0009). In the subgroup analysis, the differences were statistically significant when compared with drug treatment and ordinary acupuncture. See Figure 6 for details.

#### 3.4.3 Analysis of IgE change value

**Figure 7:**
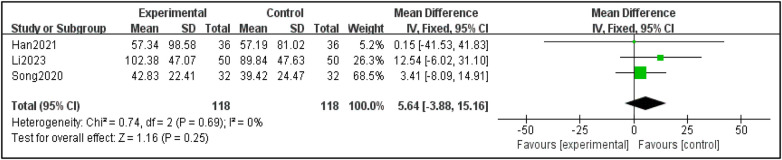
Results of the analysis of changes in IgE values before and after treatment.

A total of three studies were included in the analysis. The heterogeneity among the studies was small (I^2^ = 0%, *P* < 0.25), and the fixed effect model was used for good homogeneity. There was no significant difference in the improvement of IgE between sphenopalatine ganglion acupuncture and control group (*p* = 0.23), as shown in Figure 7.

#### 3.4.4 Total effective rate

**Figure 8:**
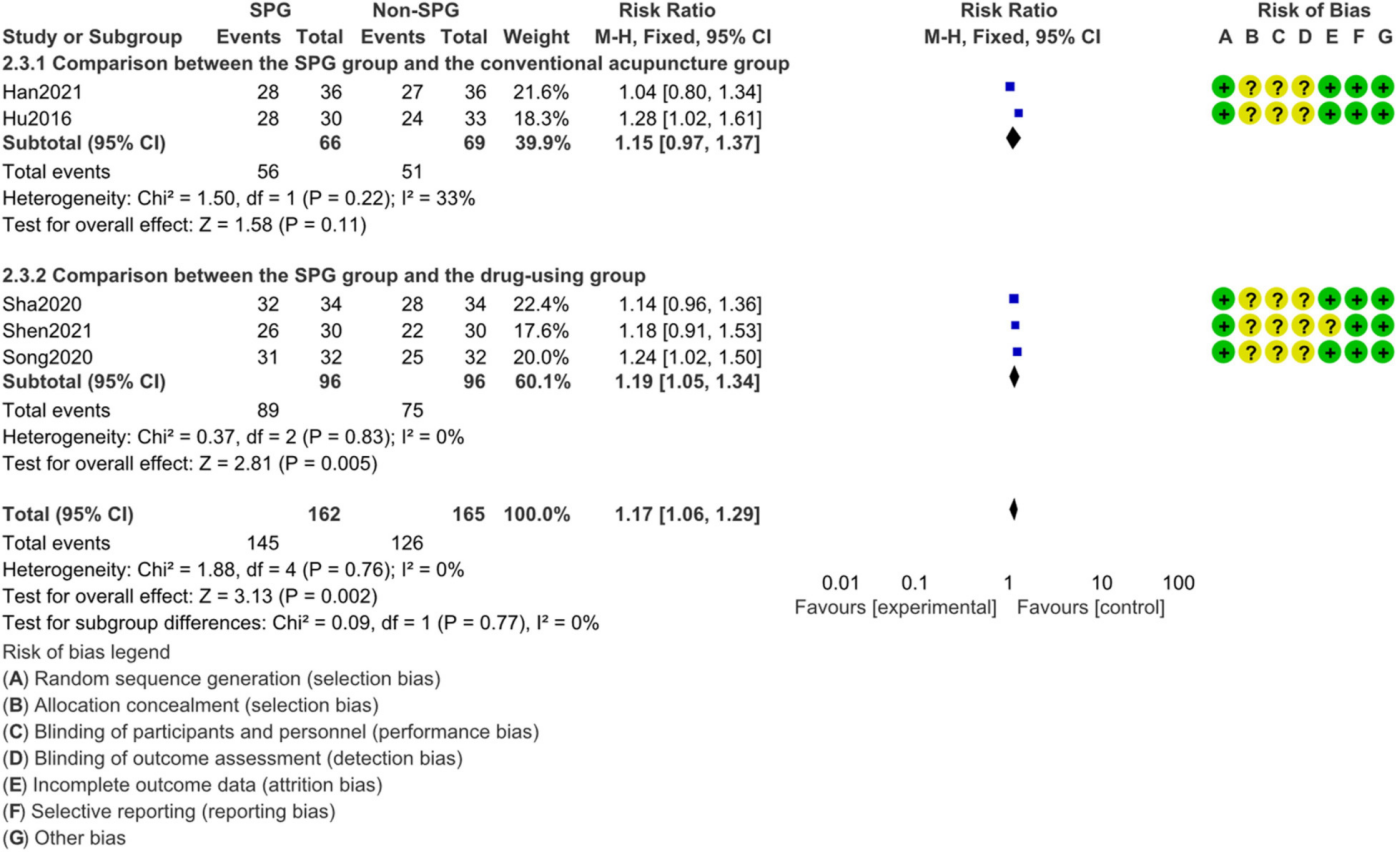
Results of total effective rate analysis (SPG:acupuncture of the pterygopalatine ganglion group, Non-SPG: the non-acupuncture ganglion of the pterygopalatine nerve group).

A total of 5 studies were included for the analysis of the total response rate. The fixed effect model was used for the analysis of the total response rate with small heterogeneity among the studies (I^2^ = 0%, *P* = 0.002). The total effective rate of sphenopalatine ganglion acupuncture in the treatment of allergic rhinitis was statistically significant (*P* < 0.002). The subgroup analysis of traditional acupuncture and drug treatment showed that acupuncture of sphenopalatine ganglion had a higher effective rate than drug treatment. As shown in Figure 8.

### 3.5 Publication bias test

**Figure 9:**
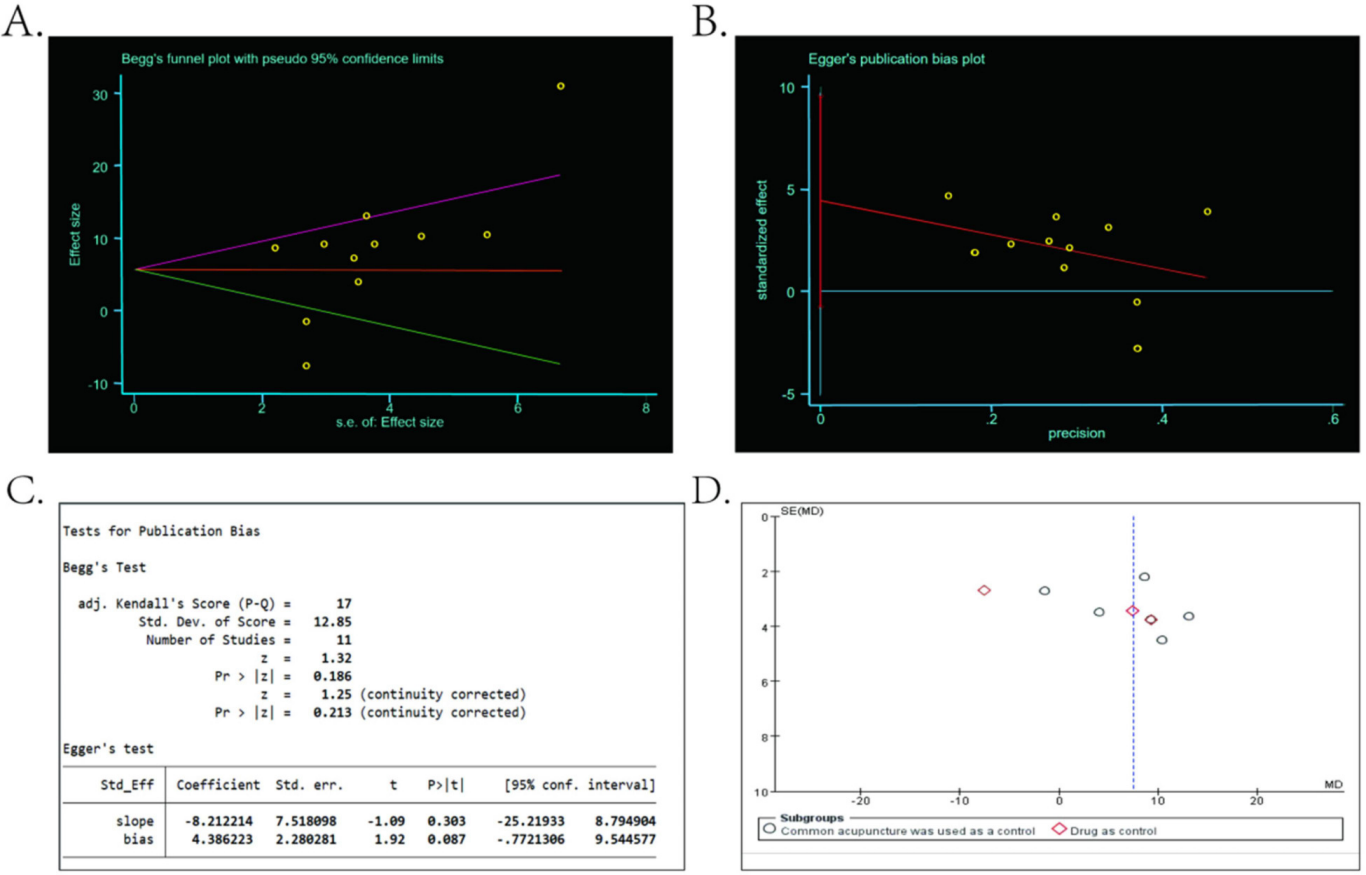
**(A)** Results of begg's test. **(B)** Egger's test results. **(C)** *P* value calculation results of Begg's test and Egger's test. **(D)** Funnel plot made using RevMan5.3.

A funnel plot was drawn according to the RQLQ scores of sphenopalatine ganglion acupuncture compared with common acupuncture and medication for allergic rhinitis, and the presence of publication bias was checked using the funnel plot, see Figure 9D for details. Due to the small number of studies, the random effects model has no auxiliary lines to help identify the presence of publication bias, and the scattered study distribution can easily be considered asymmetrical. The existence of publication bias cannot be directly determined by visual funnel plot, so StataMP17 software was used to continue the analysis, and the Egger's test and Begg's test were performed by metabias function to determine whether there was publication bias. According to the results of Egger's test and Begg's test, the 95% confidence intervals of the intercept of the two include 0, as detailed in Figures 9A,B, and the *P* values are 0.087 and 0.213, respectively, which are greater than 0.05, as shown in Figure 9C. The above results indicated that there was no significant publication bias.

### 3.6 Sensitivity analysis

**Figure 10:**
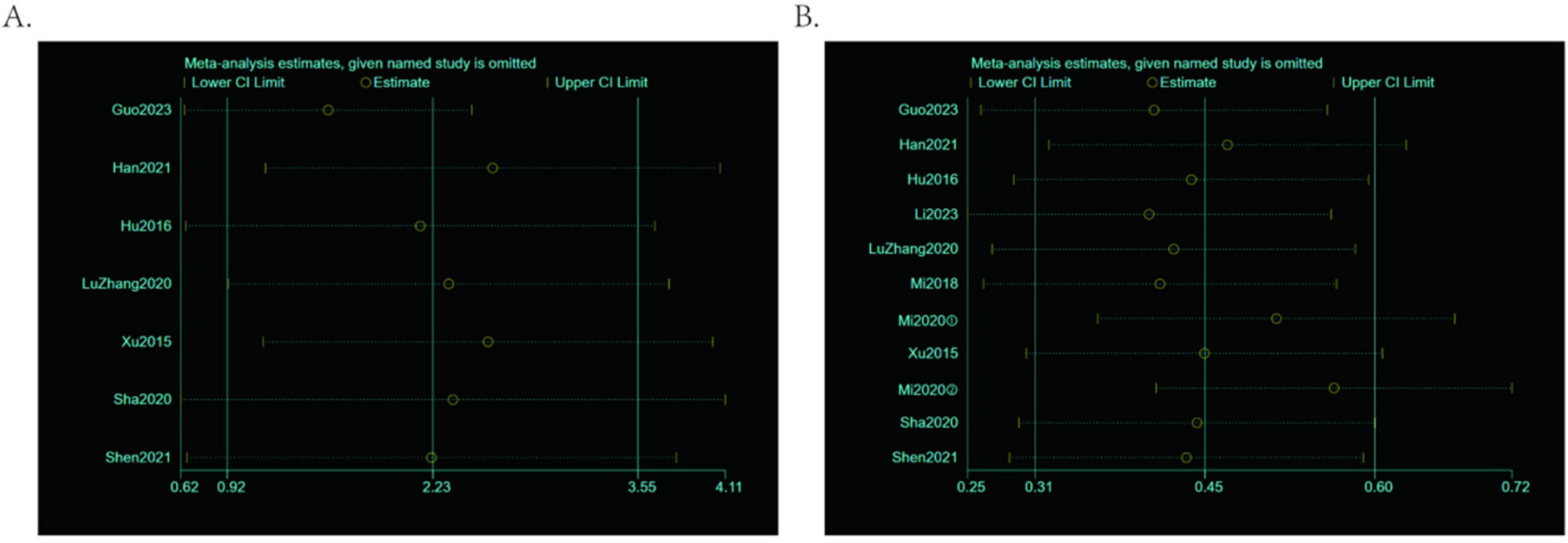
**(A)** Plot of TNSS (total nasal symptom score) sensitivity analysis results. **(B)** Plot of RQLQ (rhinoconjunctivitis quality of life questionnaire) sensitivity analysis results.

The preceding analysis obtained three statistically significant indicators: RQLQ, TNSS, and the total effective rate. Since the heterogeneity was relatively large after combining TNSS and RQLQ, the metaninf function of StataHP17 software was employed to conduct a heterogeneity analysis on the two results. Both RQLQ and TNSS are continuous variables. After successively excluding one study each time and re-examining the combined effect size, no significant changes were observed in the combined effect size, and the 95% confidence interval did not cross the null line, as shown in Figures 10A,B, indicating the stability of the results. The results of the sensitivity analysis for both did not suggest that any specific study outcome influenced the outcome indicators, still supporting that the combined effect size value of the meta-analysis was statistically significant.

### 3.7 GRADE evidence level evaluation

**Figure 11:**
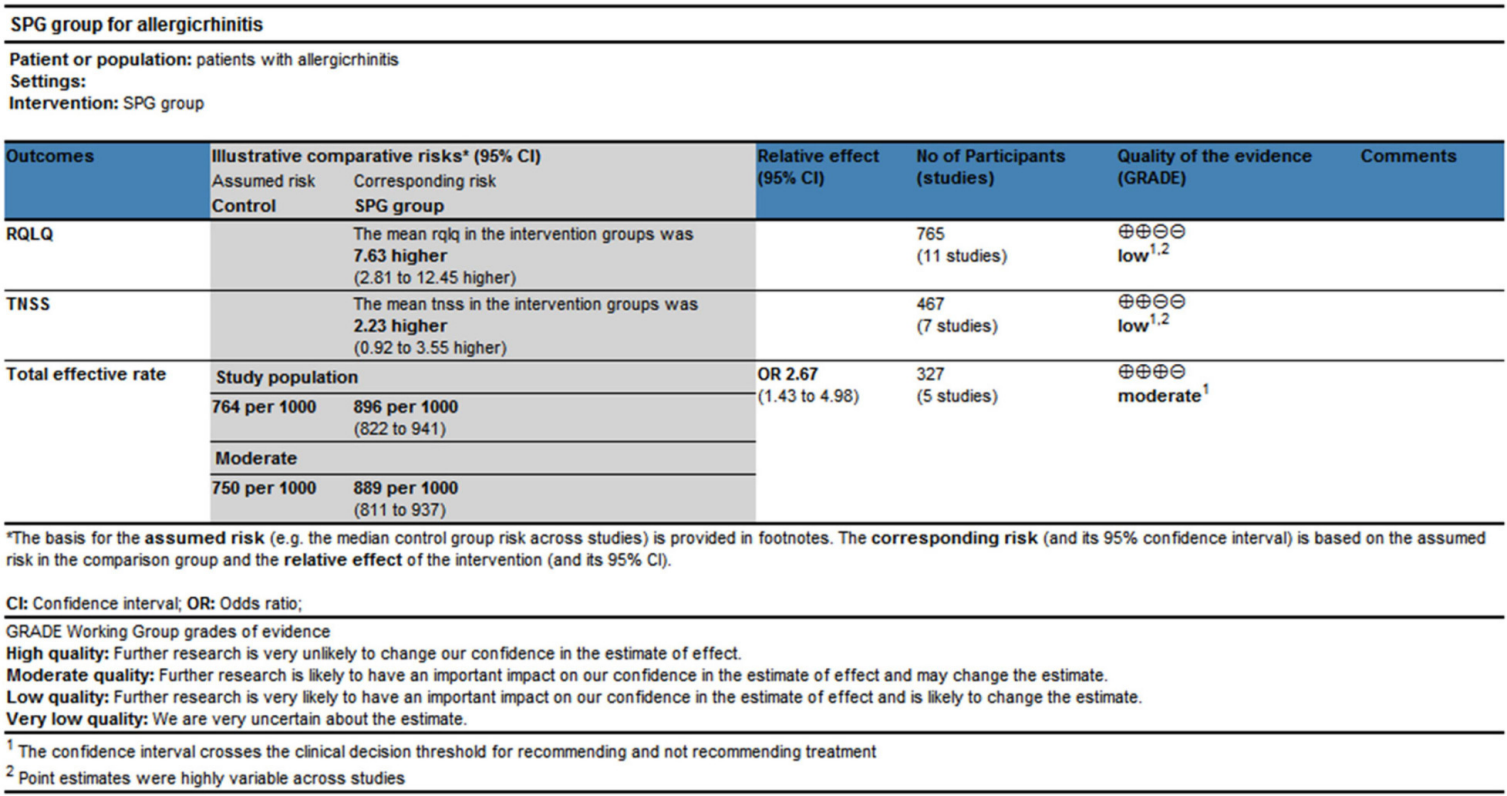
Level of evidence evaluation. (TNSS, total nasal symptom score; RQLQ, rhinoconjunctivitis quality of life questionnaire).

Three statistically significant outcome indicators were obtained from the meta-analysis, namely the changes in RQLQ score, TNSS score, and total effective rate when comparing acupuncture of the sphenopalatine ganglion with conventional Western medicine treatment and traditional acupuncture treatment. The quality of evidence was evaluated using GRADEprofiler Versions 3.6 software (26, 27). Among them, the changes in RQLQ score and TNSS score were rated as low-quality evidence, while the total effective rate was rated as moderate-quality evidence. This is shown in Figure 11.

## 4 Discussion

In the past few years, there has been a worldwide outbreak of allergic diseases, including allergic rhinitis, which are associated with a considerable medical and socioeconomic burden (2). Allergic rhinitis has become a major chronic respiratory disease (28), clinically characterized by sudden and repeated episodes of nasal itching, sneezing, runny nose, nasal congestion and other symptoms. Allergic inflammatory reactions stimulate the sensory nerves of the nasal mucosa. The classic pathway is as follows: after antigen-presenting cells first encounter external allergens, they take in information, activate T cells, and initiate a specific immune response. T cells differentiate into Th2 cells, which produce Th2-type cytokines and stimulate B cells to differentiate into plasma cells and secrete IgE. Specific IgE binds to mast cells in the nasal mucosa, initiating the release of inflammatory factors and causing a series of clinical symptoms. It can seriously affect the quality of life of patients and is an independent risk factor for asthma (28)。Its treatment (29) includes allergen avoidance, drug therapy, immunotherapy, physical therapy, surgical treatment, and other treatments (30).

The sphenopalatine ganglion is the largest parasympathetic ganglion in the head and neck. Its branches reach the superior, middle and inferior nasal conchae, nasal septum and the top of the nasopharynx, etc. It contains both parasympathetic and sympathetic nerve fibers. Acupuncture on the sphenopalatine ganglion excites the sympathetic nerve fibers in its distribution area, which is reflected to the central nervous system and transmitted to both sides of the nerves by the brain, achieving the effect of simultaneous regulation of bilateral nasal mucosa. This causes vasoconstriction, reduces blood flow in the nasal mucosa and sinuses, shrinks the volume of the nasal conchae, reduces glandular secretion, and improves ventilation and rhinorrhea. Acupuncture on the sphenopalatine ganglion can also reduce the release of vasoactive intestinal peptide and serum substance P, thereby inhibiting allergic reactions (31, 32).

This study observed significant heterogeneity in RQLQ (I^2^ = 89%) and TNSS (I^2^ = 83%), primarily stemming from the interaction of differences in intervention measures, clinical heterogeneity, and methodological limitations. In terms of intervention measures, although all employed acupuncture at the sphenopalatine ganglion (SPG), the accuracy of localization was influenced by anatomical variations and the experience of the operator, and the stimulation parameters (such as treatment course, needle retention time, and intensity) lacked standardization, leading to varying cumulative effects of neural modulation. Clinically, the included population mixed persistent/intermittent allergic rhinitis (AR), with different allergen profiles and degrees of mucosal inflammation, and some studies allowed continued use of baseline medications without monitoring compliance, further introducing confounding factors. Methodologically, RQLQ/TNSS relied on patients’ subjective recollections, with only a few studies implementing blind assessments, while others might have implementation biases, collectively amplifying heterogeneity. Therefore, the interplay of the above factors may be the main reason for the high variability in outcome indicators.

This study conducted a meta-analysis of 11 high-quality articles retrieved from multiple databases, comparing four outcome indicators: RQLQ, TNSS, total effective rate, and IgE. The research found that acupuncture at the sphenopalatine ganglion can improve the quality of life of patients with allergic rhinitis and alleviate the four main symptoms of allergic rhinitis. Limited by the number, quality of the literature and outcome indicators, the Mate data analysis still needs to be further improved. However, this does not deny the definite therapeutic effect and promotion value of acupuncture at the sphenopalatine ganglion for allergic rhinitis.

## Data Availability

The original contributions presented in the study are included in the article/Supplementary Material, further inquiries can be directed to the corresponding author.
